# Single-particle cryo-EM analysis of the shell architecture and internal organization of an intact α-carboxysome

**DOI:** 10.1016/j.str.2023.03.008

**Published:** 2023-06-01

**Authors:** Sasha L. Evans, Monsour M.J. Al-Hazeem, Daniel Mann, Nicolas Smetacek, Andrew J. Beavil, Yaqi Sun, Taiyu Chen, Gregory F. Dykes, Lu-Ning Liu, Julien R.C. Bergeron

**Affiliations:** 1 Randall Centre for Cell and Molecular Biophysics, King’s College London, London, UK; 2 Institute of Systems, Molecular and Integrative Biology, University of Liverpool, Liverpool, UK; 3 Ernst-Ruska Centre 3, Forschungszentrum Jülich, Jülich, Germany; 4 Department of Molecular Biology and Biotechnology, The University of Sheffield, Sheffield, UK; 5 College of Marine Life Sciences, and Frontiers Science Center for Deep Ocean Multispheres and Earth System, Ocean University of China, Qingdao, Shandong, China

**Keywords:** cryo-EM, carboxysome, photosynthesis, cyanobacteria

## Abstract

Carboxysomes are proteinaceous bacterial microcompartments that sequester the key enzymes for carbon fixation in cyanobacteria and some proteobacteria. They consist of a virus-like icosahedral shell, encapsulating several enzymes, including ribulose 1,5-bisphosphate carboxylase/oxygenase (RuBisCO), responsible for the first step of the Calvin-Benson-Bassham cycle. Despite their significance in carbon fixation and great bioengineering potentials, the structural understanding of native carboxysomes is currently limited to low-resolution studies. Here, we report the characterization of a native α-carboxysome from a marine cyanobacterium by single-particle cryoelectron microscopy (cryo-EM). We have determined the structure of its RuBisCO enzyme, and obtained low-resolution maps of its icosahedral shell, and of its concentric interior organization. Using integrative modeling approaches, we have proposed a complete atomic model of an intact carboxysome, providing insight into its organization and assembly. This is critical for a better understanding of the carbon fixation mechanism and toward repurposing carboxysomes in synthetic biology for biotechnological applications.

## Highlights

• Carboxysomes are prokaryotic microcompartments that sequester photosynthetic enzymes
• We used single-particle cryo-EM analysis to characterize intact carboxysome complexes
• We have proposed a model of how enzymes are arranged within carboxysomes
• We also have generated a structural model of its shell architecture

## Graphical abstract

**Figure:**
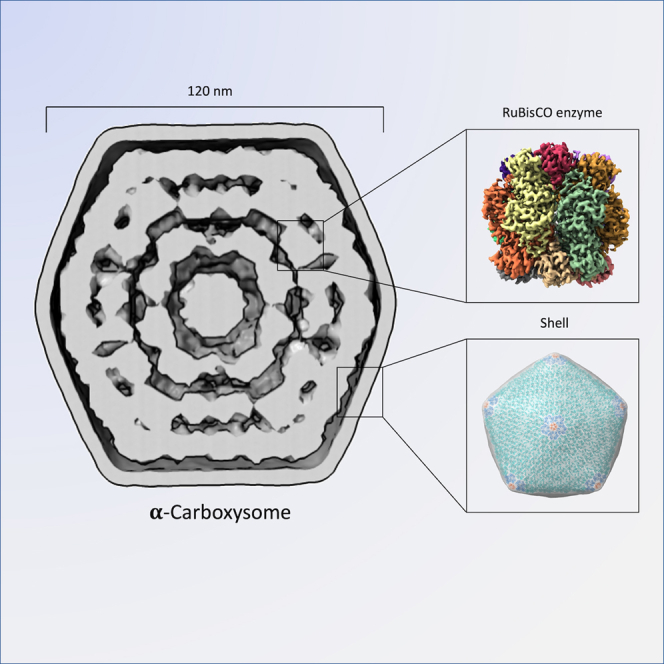

## Teaser

Carboxysomes are proteinaceous microcompartments found in cyanobacteria, which facilitate carbon fixation, the first step of photosynthesis, by sequestering the corresponding enzymes. Evans et al. used cryoelectron microscopy to characterize intact carboxysomes, which allowed them to establish a structural model for the carboxysome shell and for its internal enzyme organization.

## Introduction

Within cells, proteins tend to self-assemble and interact with other proteins or molecules to form active macromolecular machines,^1^^,^^2^^,^^3^ which play central roles in many cellular processes.^4^^,^^5^ Understanding the precise structures of natural protein assemblies is imperative for fundamental investigations of their biosynthesis and functions, and towards engineering artificial nanomaterials for new functions.^6^

Bacterial microcompartments (BMCs) are large macromolecular assemblies widespread in the bacterial domain.^7^^,^^8^ BMCs serve as metabolic organelles, but unlike their eukaryotic counterparts, they have no lipid bilayer and are composed entirely of proteins.^8^ By segregating metabolic enzymes from the cytosol, BMCs are thought to protect the cell from toxic intermediate metabolites and unwanted side reactions, and play pivotal roles in several enzymatic pathways, including autotrophic CO_2_ fixation and catabolic processes.^9^^,^^10^^,^^11^^,^^12^

Despite their diverse range of functions, all BMCs possess a similar overall organization. They consist of a polyhedral proteinaceous shell, reminiscent of viral capsids. This shell encapsulates the enzymes involved in the corresponding metabolic pathway and acts as a semi-permeable physical barrier for molecule diffusion.^1^^,^^13^^,^^14^ Structural studies of multiple BMC shell proteins in isolation have shown that they belong to three distinct categories: hexamers and pseudo-hexameric trimers, which tile the majority of the shell facets, and pentamers, which cap the vertices of the polyhedral shell.^2^^,^^15^^,^^16^^,^^17^ Although our knowledge about the entire architecture of BMCs is still primitive, high-resolution cryoelectron microscopy (cryo-EM) structures of synthetic BMC minishells have provided insight into the organization of shell proteins and the dynamic nature of these proteinaceous shells for facilitating metabolite entry and exit.^18^^,^^19^^,^^20^

Carboxysomes were the first BMCs to be discovered.^21^ They are found in cyanobacteria and some chemoautotrophs, and play a key role in carbon fixation.^22^ Carboxysomes contain the enzymes ribulose-1,5-bisphosphate carboxylase/oxygenase (RuBisCO) and carbonic anhydrase (CA). CA catalyzes the conversion of cytosolic bicarbonate (HCO_3_^−^) into CO_2_, which is subsequently utilized by RuBisCO and fixed onto the 5-carbon sugar ribulose-1,5-bisphosphate (RuBP) as the first step in the Calvin-Benson-Bessham (CBB) cycle.^23^ By generating carboxysomes to sequester these enzymes and allow the accumulation of HCO_3_^−^/CO_2_, bacterial cells can provide an elevated level of CO_2_ around RuBisCO to enhance carbon fixation and overcome the competitive inhibition of RuBisCO carboxylation by O_2_.^22^^,^^24^ These intrinsic structural features allow carboxysomes to make a significant contribution to the global carbon fixation.^22^ Notably, repurposing carboxysomes is an emerging discipline with applications in crop engineering, metabolic enhancement, bioenergy production, and therapeutics.^22^^,^^25^^,^^26^^,^^27^

Carboxysomes can be classified into two distinct groups: α-carboxysomes, primarily encoded for by the *cso* operon, and β-carboxysomes, primarily encoded for by the *ccm* operon.^28^ These two groups are distinct due to their protein composition and the types of RuBisCO encapsulated, belonging to form 1A and form 1B RuBisCO, respectively. Despite having been suggested to have evolved independently to adapt to different ecological niches, these two forms of RuBisCO demonstrate similar affinities for their substrates.^29^ Unlike RuBisCO and shell proteins, the CA enzyme is largely evolutionarily distinct between α-carboxysomes and β-carboxysomes. CsoSCA possesses a distinct fold from CcmM, the CA enzyme found in β-carboxysomes (and some α-carboxysomes), although both proteins are essential for function, and both carry out similar roles.^30^

RuBisCO is a hexadecameric complex, comprised of eight large subunits and eight small subunits. The structures of RuBisCO from various cyanobacteria and plant species have been solved.^31^^,^^32^^,^^33^^,^^34^^,^^35^ In β-carboxysomes, RuBisCO enzymes appear densely organized and form paracrystalline arrays that are important for β-carboxysome biogenesis.^36^^,^^37^^,^^38^^,^^39^ In contrast, RuBisCO enzymes have been postulated to assemble concomitantly with the shell during α-carboxysome biogenesis, a process promoted by the intrinsically disordered protein CsoS2, which induces the association between shell proteins and interiors.^17^^,^^40^ The organization of RuBisCO inside α-carboxysomes is poorly understood. Previous cryo-electron tomography analysis of α-carboxysomes from the chemoautotrophic bacterium *Halothiobacillus neapolitanus,* and the cyanobacterial strains *Prochlorococcus marinus* MED4 and *Synechococcus* sp. WH8102 and WH8109, showed that the RuBisCO and CA enzymes appear to be packed densely and arranged into concentric layers.^41^^,^^42^^,^^43^^,^^44^ However, no model has been proposed for the protein arrangement and interactions within the carboxysome and the architecture of the carboxysome shell. X-ray laser single-particle diffraction outlined the icosahedral shape of the α-carboxysomes from *Halothiobacillus neapolitanus*, but no high-resolution structures of the intact carboxysome and the interior organization were reported.^45^ These studies, together with those of other BMCs, have highlighted the challenges associated with structural characterization of these large heterogeneous macromolecular assemblies, specifically with great variations in the stoichiometric composition and interactions of individual building components that are adaptive to environmental changes.^20^^,^^36^^,^^46^

Here, we report the single-particle cryo-EM analysis of an intact α-carboxysome from the marine α-cyanobacterium *Cyanobium* sp. PCC 7001 (hereafter *Cyanobium*). We have determined the structure of its RuBisCO enzyme to 2.9 Å resolution, with the densities present for the substrate RuBP and for an unknown ligand. We also report a low-resolution structure of the icosahedral shell, demonstrating a range of dimensions, which precludes high-resolution analysis but nonetheless allows us to propose a hybrid structural model for the α-carboxysome shell architecture. Moreover, 3D reconstruction combined with modeling allows us to propose a model for the arrangement of RuBisCO enzymes within the α-carboxysome. The study provides insight into α-carboxysome assembly, which will inform rational design and engineering of BMC-based nanostructures for diverse purposes.

## Results

### Purification and single-particle analysis of α-carboxysomes from *Cyanobium*

**Figure 1:**
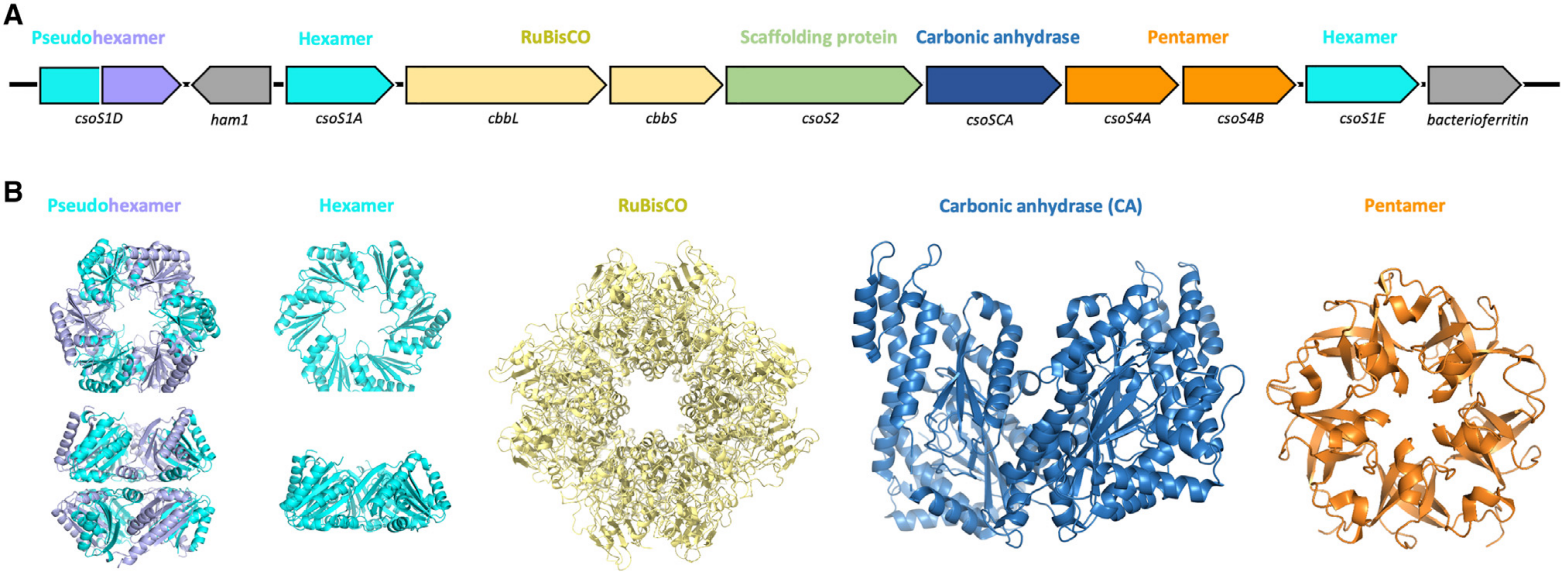
The *Cyanobium* sp. PCC7001 α-carboxysome (A) Gene organization of the α-carboxysome operon, including genes encoding the shell hexamers (cyan) and pentamers (orange), the scaffolding protein (green), and the cargo enzymes RuBisCO (yellow) and CA (blue). (B) Structural models of the corresponding α-carboxysome proteins, based on the previously determined structures of homologous proteins.

The *Cyanobium* α-carboxysome is encoded by a 9-gene operon, including 5 genes corresponding to shell proteins (*csoS1D*, *csoS1A*, *csoS4A*, *csoS4B*, and *csoS1E*), 3 genes encoding cargo enzymes RuBisCO (*cbbL* and *cbbS*) and CA (*csoSCA*), and 1 gene encoding the scaffolding protein CsoS2 (*csoS2*) (Figure 1A). CsoS1A and CsoS1E contain one Pfam00936 domain, homologous to the prototypical BMC shell hexamer, that tiles the majority of the α-carboxysome shell; in addition, CsoS1E also possesses ∼80 residues at its N terminus that are predicted to be unstructured. CsoS1D, containing two fused Pfam00936 domains, shows similarity to pseudo-hexamer trimers (sequence identity: 67%), corresponding to two stacked hexamers and presumably responsible for the passage of large molecules in and out of the carboxysome (we note, however, that the formation of pseudo-hexamers for this protein has not been confirmed experimentally). CsoS4A and CsoS4B have one Pfam03319 domain and belong to the family of BMC shell pentamers that cap the vertices of the polyhedral shell (Figure 1B).

**Figure 2:**
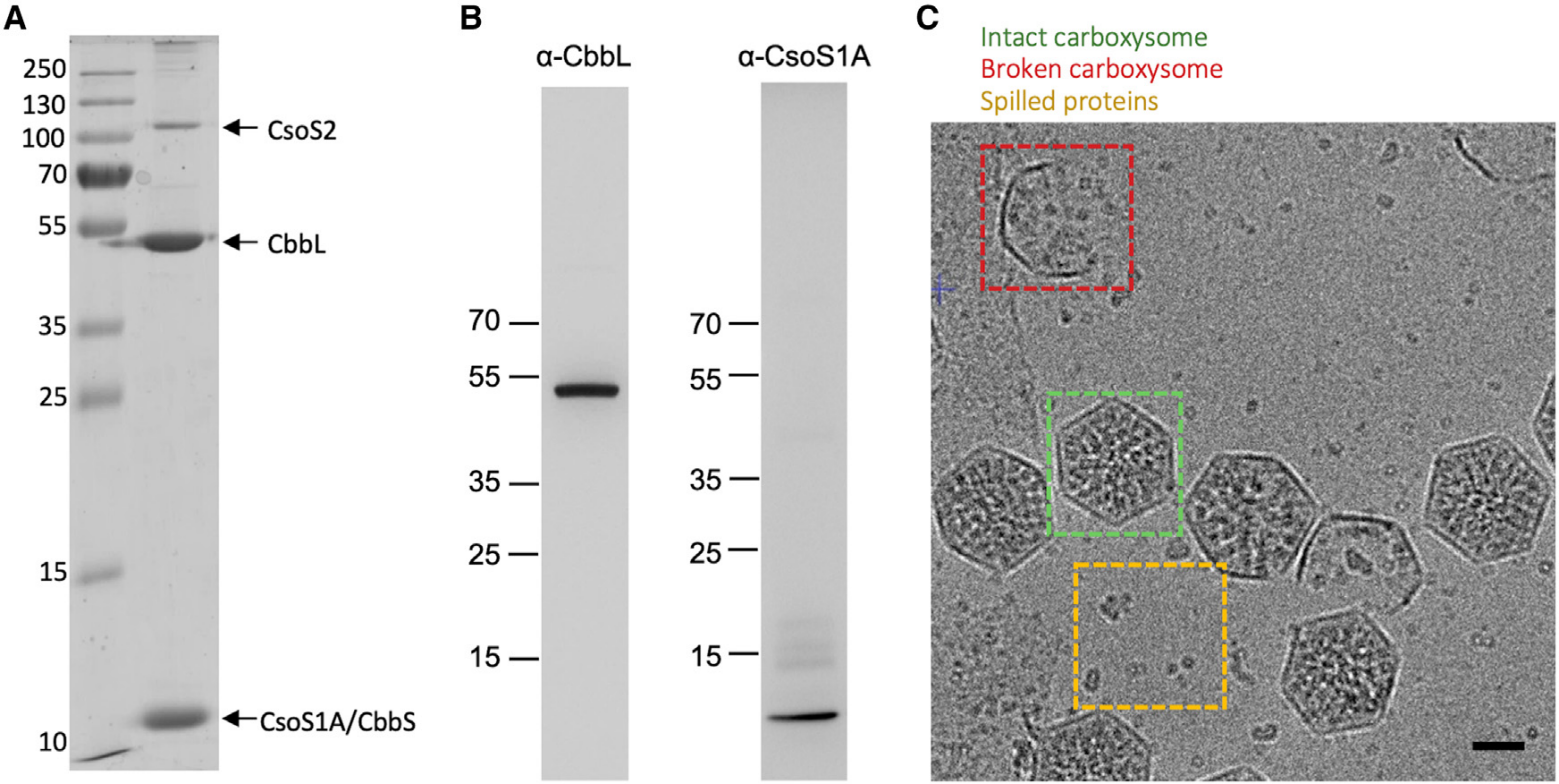
Purification and cryo-EM analysis of the *Cyanobium* α-carboxysome (A) SDS-PAGE of the purified carboxysomes. Bands for proteins CsoS2, CbbL, CbbS, and CsoS1A could be identified. (B) Western blotting of the purified α-carboxysome complex, using antibodies raised against peptides from CbbL and CsoS1, confirming the presence of both proteins. (C) Cryo-electron micrograph of frozen-hydrated α-carboxysome samples. Intact BMCs, with incorporated proteins, are visible (green box), along with broken ones (red box). Smaller protein complexes, presumably spilled from these, are also visible (yellow box). Scale bar: 50 nm. See also Figure S1 and Table S1.

To isolate functional carboxysomes, we grew *Cyanobium* photosynthetically in BG-11 freshwater medium until the late exponential phase (Figure S1A). Native α-carboxysomes were isolated from *Cyanobium* using sucrose gradient ultracentrifugation and were enriched at the 30%–40% sucrose fraction (Figure S1B). SDS-PAGE (Figure 2A) and immunoblot analysis (Figure 2B) of the 40%–50% fraction demonstrated the presence of major α-carboxysome components CbbL, CsoS2, and CsoS1A. Mass spectrometry analysis further indicated that the isolated α-carboxysomes comprise all 9 building proteins (Table S1). Among them, RuBisCO subunits (CbbL and CbbS), CsoS2, and CsoS1A are highly abundant proteins, while CsoS4A, CsoS4B, and CsoS1D have low abundance in the α-carboxysome, in good agreement with the mass spectrometry data of α-carboxysomes from *Halothiobacillus neapolitanus*.^47^ Nonetheless, due to the large errors for these measurements, the exact stoichiometry for each component could not be established unambiguously. Negative-stain EM showed that the isolated α-carboxysomes have a canonical polyhedral BMC shape, with an average diameter of ∼120 nm (Figure S1C), comparable to previous observations.^29^^,^^48^ A ^14^C-based assay of RuBisCO activity confirmed that the isolated α-carboxysomes are catalytically active for carbon fixation (Figure S1D).

These intact, functional α-carboxysomes were then subjected to single-particle cryo-EM analysis to study their 3D architecture. Initial screening showed a heterogeneous sample, containing intact carboxysomes with proteinaceous content, but also broken carboxysome shell fragments without any cargo inside, and disassembled proteins outside of the carboxysomes (Figure 2C). This deviates from negative-stain EM results (Figure S1C). We postulate that the broken shells largely result from sample handling and/or freezing, and that the disassembled proteins correspond to the proteins that spilled from the broken carboxysomes, including the enzymes RuBisCO and CA, as well as isolated shell components.

### Structure of RuBisCO from native α-carboxysomes

To gain structural insights into the α-carboxysomes, we collected a cryo-EM dataset of the sample described above using a standard, high-magnification (∼1 Å/pix^2^) data collection approach. Because of the size of the complex, and its propensity to break (see above), we only obtained a few intact carboxysomes fully visible within each micrograph in this dataset. This largely precluded any analysis of the carboxysome complex. However, the spilled particles were readily visible on ice, and we were able to pick these, leading to a set of ∼3,000,000 particles.

Following initial 2D classifications, clear classes of two distinct molecular species could be identified. Specifically, several classes showed clear 4-fold symmetry and were visually identified as the RuBisCO (CbbL_8_-CbbS_8_) holoenzyme (Figure S2A). Additional classes were obtained for smaller protein(s), but these were featureless, and the corresponding protein(s) could not be identified based on 2D classes (Figure S2B). We hypothesize that these proteins correspond to a mixture of CA and shell proteins; however, this would require further validation.

**Figure 3:**
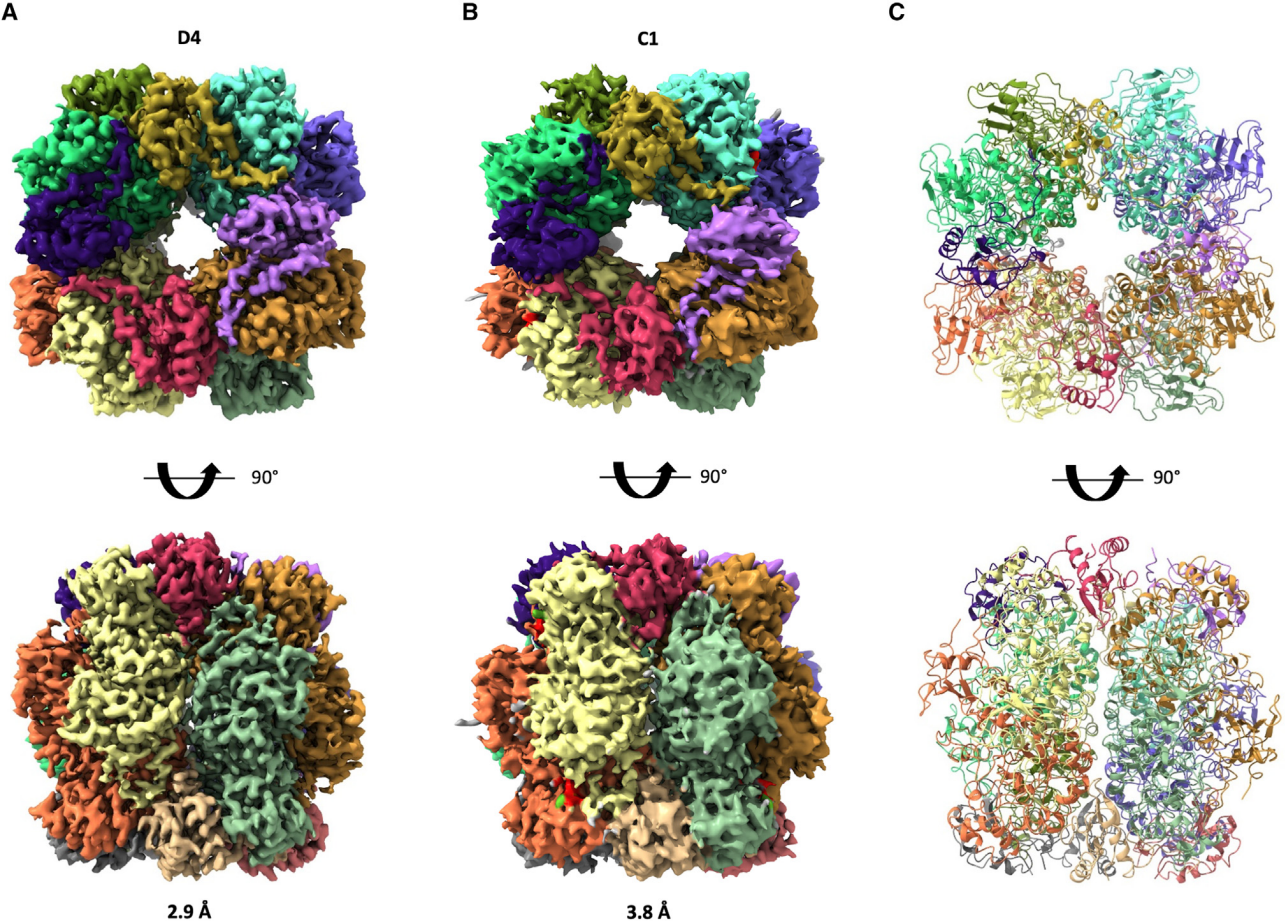
Cryo-EM structure of RuBisCO isolated from *Cyanobium* α-carboxysomes (A and B) The coulomb potential map of RuBisCO, obtained from the particles of spilled RuBisCO proteins, colored and segmented by chain, is shown for the map with D4 symmetry (A) and without symmetry (B). (C) Atomic model of the *Cyanobium* RuBisCO enzyme, in cartoon representation, and colored as in (A) and (B). See also Figures S2–S4 and Video S1.

We next conducted 3D refinement using the set of particles that could be identified as RuBisCO in the 2D classes. This yielded a 2.9 Å resolution coulomb potential map (Figures 3A, S2C, and S2E; Table 1; Video S1), with eight large subunits (CbbL) and eight small subunits (CbbS) readily identifiable. Using this map, we were able to build an atomic model of the *Cyanobium* RuBisCO enzyme (Figure 3C; Table 1). Notably, density is present in the active site, in a position suitable to be the substrate RuBP and magnesium ion, which allowed us to model these molecules in the active site (Table 1). This observation demonstrates that most RuBisCO enzymes within the carboxysome are active and bound to the substrates, in agreement with the RuBisCO assay results (Figure S1D).

**Table 1:** Cryo-EM data collection and structure refinement parameters

	Dataset 1		Dataset 2
**Data collection**			

Voltage (kV)	300		300
Exposure (e/Å^2^)	30		29.7
Fractions	44		33
Defocus range (μm)	−0.5 to −1.5		−1 to −2.2
Pixel size (Å pix^−1^)	1.11		2.23
No. of micrographs	4,593		5,429
Initial particle no.	2,800,000		15,545

**Map refinement**			

Final particle no.	131,356	131,356	3,533
Resolution (Å)	2.87	3.79	18.25
Symmetry	D4	C1	I

**Structure refinement**			

Non-hydrogen atoms	35,138	35,070	–
Protein residues	4,432	4,424	–
Ligands	16	10	–
Protein B factor	30.19	81.71	–
Ligand B factor	68.53	113.28	–
Bond length RMSD (Å)	0.007	0.002	–
Bond angle RMSD (°)	0.790	0.668	–
MolProbity score	2.02	1.78	–
Clash score	8.23	11.83	–
Poor rotamers	2.51	0.11	–
Ramachandran favored (%)	96.11	96.81	–
Ramachandran allowed (%)	3.70	3.03	–
Ramachandran disallowed (%)	0.18	0.16	–

RMSD, root-mean-square deviation.

Video S1. Structure of the *Cyanobacterium* sp. RuBisCO, related to Figure 3

However, we note that this density for the substrates is at a significantly lower contour level compared with the protein density, indicative of partial occupancy. To resolve which subunit(s) contained the substrate, we performed a refinement of the RuBisCO map without imposing any symmetry, leading to a second map at 3.8 Å resolution (Figure 3B; Table 1). As shown on Figure S3, we observed varied levels of density in all eight active sites: for one chain, the full RuBP density can be readily identified (chain G), whereas in two chains (A and E), no density is present at all. Finally, we observe partial substrate density in five chains (C, I, K, M, and O), which could correspond to one or two molecules of the reaction product, 3-phosphoglycerate, and/or a mix of states that are averaged out through alignment. This confirms the activity of the RuBisCO enzymes present in carboxysome, with various stages of the reaction cycle present within one different subunit of the same complex. While we do not think that there is specific cooperativity between the eight subunits of the complex, this might indicate some correlation in the catalytic reaction between subunits, perhaps regulated by interacting proteins such as CA and/or CSoS2.

To further investigate the catalytic state of the RuBisCO complex, we analyzed the conformation of the large subunit loop 6, which had previously been shown to adopt two distinct conformations, corresponding to apo (open) or substrate-bound (closed) states of the enzymes.^32^^,^^49^ In our RuBisCO map, the density of this loop is poorly resolved, suggesting that it is likely dynamic. Nonetheless, we were able to build an atomic model for the entire loop. When compared with the crystal structures of the spinach RuBisCO (71% identity to CbbL, 29% to CbbS) in both open and closed states, we observed that on our map, this loop corresponds to the open conformation (Figure S4A), likely providing an explanation for the range of substrate-/product-bound states observed (see above).

We also note that in the map obtained in the absence of symmetry, some unattributed, diffuse density at a lower contour level is present at the surface of the complex for one of the subunits (Figure S4B). This suggests that, for some of the particles, other proteins are bound in this location. This region of the map is at a much lower resolution and did not allow us to identify what protein this might be based on the density alone. Nonetheless, this finding provides evidence that there are other proteins bound to RuBisCO, which likely originates from the broken carboxysomes. Further investigation is required to determine which carboxysomal protein the density represents. We note, nonetheless, that this density is not in the region of the complex where CsoS2 had previously been shown to bind, suggesting that either CsoS2 has multiple modes of interaction with RuBisCO or that this density corresponds to a different protein.^40^

### Cryo-EM analysis of the α-carboxysome shell

The current structural information on α-carboxysomes is limited to low-resolution tomography data.^41^^,^^42^^,^^44^^,^^50^^,^^51^ We therefore attempted to use single-particle cryo-EM to gain insight into the overall architecture of the *Cyanobium* α-carboxysome shell. As mentioned above, both the manual handling and freezing of the complex led to significant breakage, which prevented large data collection of intact carboxysomes. To address this, we froze grids immediately after purification, leading to a higher proportion of intact carboxysomes. In addition, we collected data at lower magnification (Table 1), allowing a larger field of view to include more intact particles. Collectively, these strategies allowed us to collect a second dataset with an average of 2–3 intact complexes per micrographs, leading to a set of 15,545 shell particles.

Initial 2D classification of the intact carboxysome complexes was carried out (Figure S5). In these 2D classes, the cargos within the carboxysome shell are clearly ordered and organized into concentric layers, in line with the findings from previous α-carboxysome studies by electron tomography.^42^^,^^44^ 3D refinement attempts with this set of particles, without symmetry, failed to converge to interpretable models, with all the particles clustered in a small subset of angle assignments. We therefore carried out a masked 3D classification selectively for the shell (Figure S6), with icosahedral symmetry applied. This led to several classes of particles, of varying diameters from 119 to 123 nm (Figure S6), demonstrating the size heterogeneity of the *Cyanobium* α-carboxysomes.

We next performed 3D refinement on the most populated class of particles, applying icosahedral symmetry with masking of the internal density. This led to a map of the carboxysome shell at ∼18 Å resolution (Figures 5A and S6B). At this resolution, the map is largely featureless but still allows us to clearly identify the edges of the icosahedron. We also note that previous studies on synthetic BMC shells have revealed that some pseudo-hexamers form double-layered complexes that protrude from the shell surface.^20^^,^^52^ Such protrusions made of the pseudo-hexamers CsoS1D were not visible on our reconstruction, which could indicate that it is not the case for CsoS1D. Alternatively, it is possible that CsoS1D is distributed randomly on the shell surface, and therefore double layers are blurred out during reconstruction. A higher-resolution map, obtained without symmetry, would be required to verify this.

**Figure 4:**
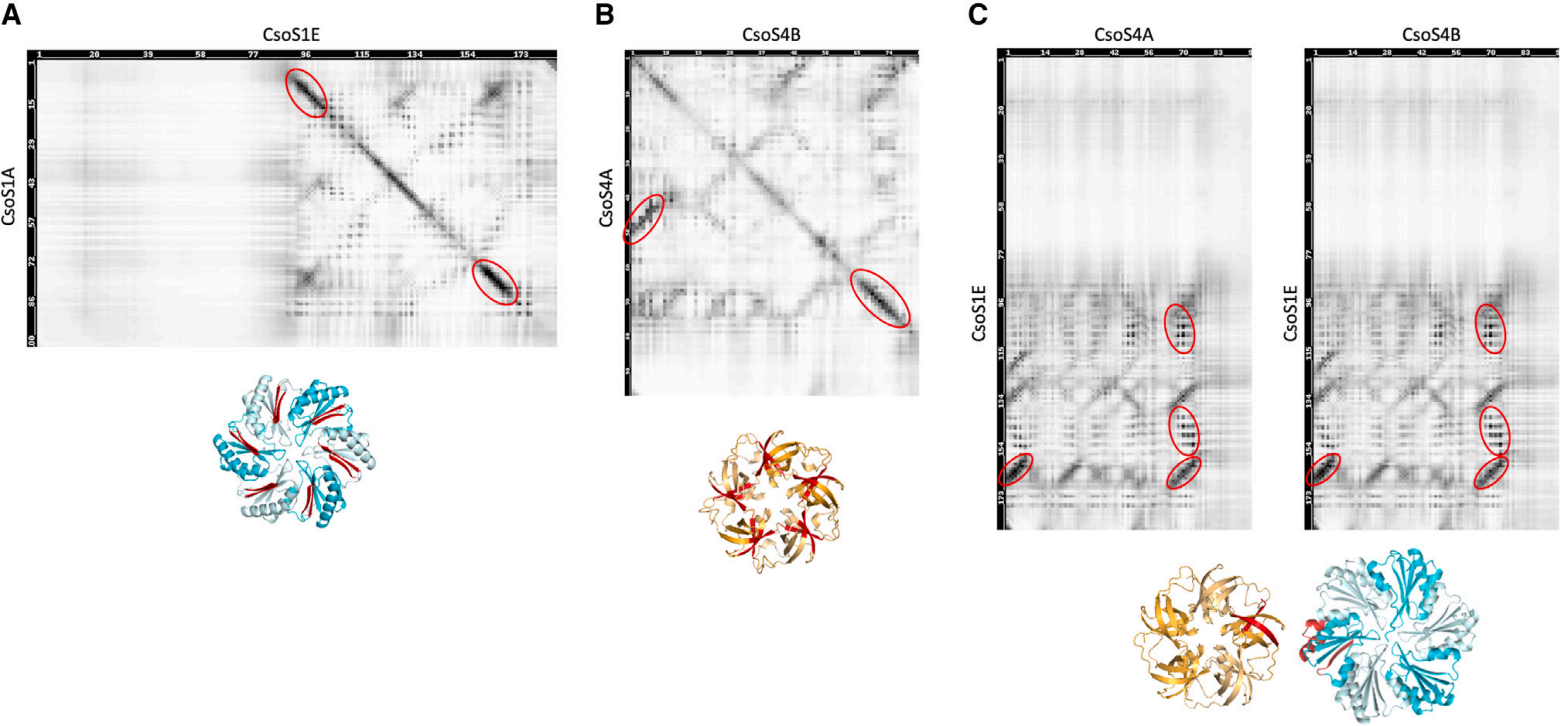
Co-evolution maps of the α-carboxysome shell proteins For each protein pair, the co-evolution map is shown at the top, and an atomic model is at the bottom, colored as in Figure 1. The residues with strong co-evolution correlation are indicated with a red circle and colored in red in the structural model. The co-evolution analysis for CsoS1A and CsoS1E (A), as well as CsoS4A and CsoA4B (B), strongly suggests interhomooligomer interactions. In contrast, in the case of CsoS1E and CsoS4A/B (C), intrahomooligomer interactions are likely identified. See also Table S2.

### Modeling of the α-carboxysome shell architecture

As indicated above, the resolution of the map of the α-carboxysome shell is not sufficient to build an atomic model *de novo*. Nonetheless, we used a hybrid approach, by combining this map with the previously elucidated structures of shell proteins and other modeling tools, to propose a structural model of the *Cyanobium* α-carboxysome shell.

Specifically, we used co-evolution analysis (see STAR Methods for details) to determine the interactions between various shell proteins. We found a strong co-evolution correlation between CsoS1A and CsoS1E and between CsoS4A and CsoS4B (Table S2; Figures 4A and 4B). Mapping the regions with the strongest co-evolution links on the atomic models revealed that they correspond to the homo-oligomer interface (Figures 4A and 4B). These results suggest that α-carboxysome shell proteins have strong tendencies to form hetero-oligomers, i.e., hexamers formed by a combination of CsoS1A and CsoS1E, and pentamers formed of both CsoS4A and CsoS4B, as demonstrated previously in β-carboxysomes.^53^^,^^54^

In addition, we observed a strong co-evolution correlation between CsoS1E and both CsoS4A and CsoS4B (Figure 4C). In contrast, the correlation between CsoS1A and CsoS4A/B was very limited (Table S2). This suggests that the interaction between hexamers and pentamers is formed specifically by CsoS1E, forming the first layer of hexamers around pentamers, while CsoS1A forms predominantly hexamer-hexamer interactions. We note that, alternatively, it is possible that this layer consists of a CsoS1E-CsoS1A hetero-oligomer, with CsoS1E exclusively at the interface with CsoS4A/B.

**Figure 5:**
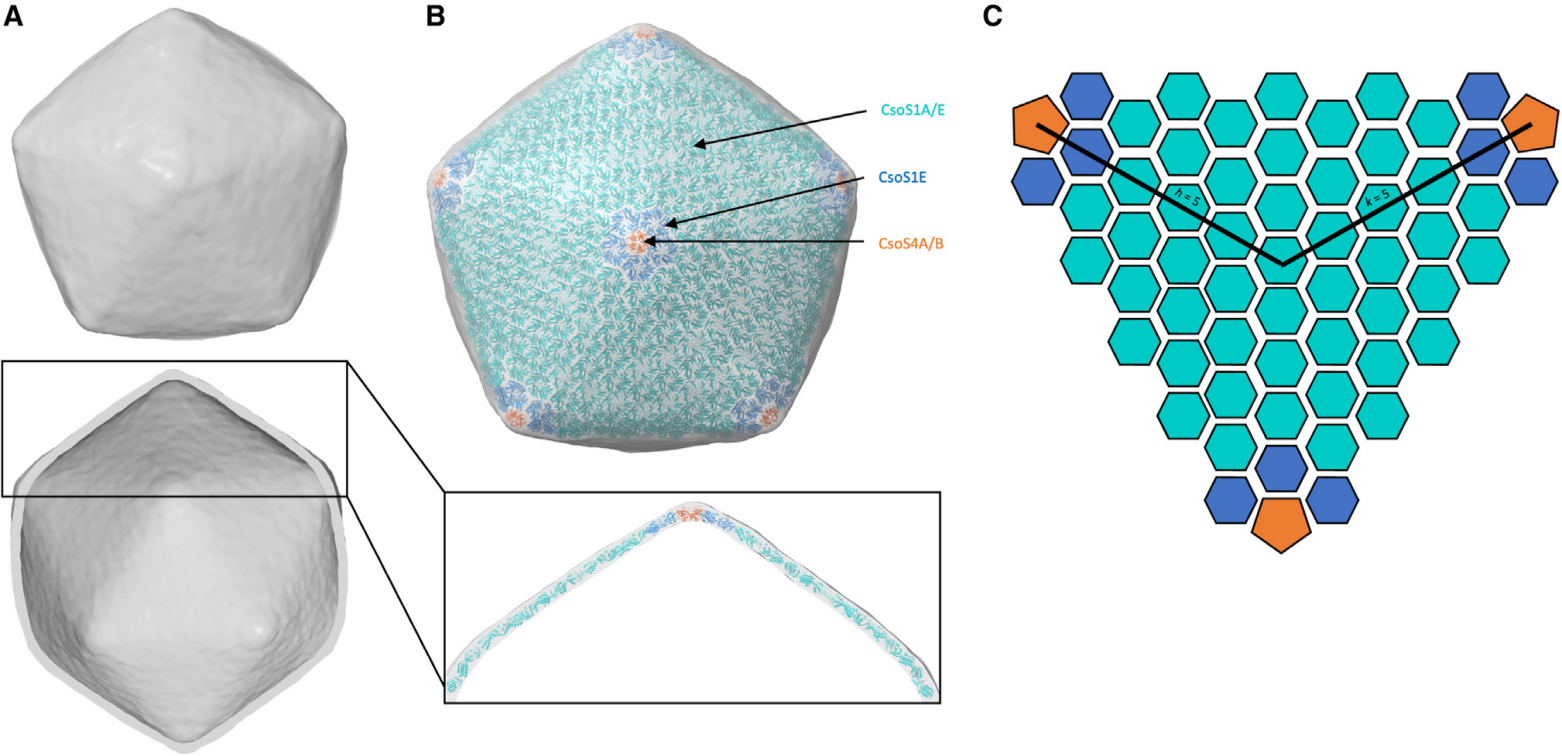
Architecture of the α-carboxysome shell (A) Electron potential map of the carboxysome shell, to ∼18 Å resolution. (B) Atomic model of the carboxysome shell. Pentamers (likely a mixture of CsoS4A and CsoS4B), located at the vertices of the shell, are shown in orange. They are surrounded by five dimers, probably consisting mostly of CsoS1E, in blue. Additional hexamer layers form the remaining icosahedral face, formed predominantly by CsoS1A, in cyan. (C) Schematic representation of one face of the carboxysome shell model, colored as in (B). Black lines indicate the unit count for calculation of the T number. See also Figure S5, and Video S2.

We next combined the hexamer and pentamer orientation derived from the previous structure of a synthetic β-carboxysome shell,^16^ the low-resolution map of the *Cyanobium* α-carboxysome shell (Figure 5A), and the co-evolution data (Table S2; Figure 4) to build an atomic model of the intact α-carboxysome shell (Figure 5B; Video S2). In this model, the α-carboxysome shell is comprised of 12 pentamers and 750 hexamers. This corresponds to a triangulation (T) number of 75 (*h* = 5; *k* = 5; T = *h*^2^ + *hk* + *k*^2^) (Figure 5C).^55^ As indicated above, there is variation in the dimensions of the shell, which likely corresponds to breathing between shell subunits. Further structural analysis, using a much larger number of intact α-carboxysome particles, is required to verify this interpretation.

Video S2. Structural model of the *Cyanobacterium* sp. carboxysome shell, related to Figure 5

Intriguingly, we also observed a very limited co-evolution correlation between CsoS1D and any other shell proteins (Table S2). This could partially be due to its low abundance in the shell,^47^ in agreement with SDS-PAGE and mass spectrometry analysis (Figure 2; Table S1), but likely indicates that it is randomly localized in the shell facets. As such, CsoS1D is not included in this structural model. However, this protein was explicitly present within the α-carboxysome (Table S1). The role and position of CsoS1D within the shell merit further characterization.

### Internal arrangement of enzymes within the α-carboxysome

To further characterize the internal organization of the α-carboxysomes, we carried out masked 3D refinement on the internal density (Figure S6). We initially attempted reconstructions using a range of symmetries (Figure S7); however, in most cases, this led to the blurring and distortion of features in the obtained maps. Subsequently, we applied masked 3D icosahedral refinements of individual rings of densities observed within the carboxysomes. These yielded several reconstructions to ∼18 Å, with continuous density for each layer, which we termed the outmost, middle, inner, and core layers, respectively (Figures S6A and S6B). Notably, all these layers are of a thickness that is similar to the height of RuBisCO (∼10 nm) and possess discernible features that broadly resemble RuBisCO’s shape. We note, however, that features with 3- and 5-fold symmetry are present in this map but are likely artifacts of the imposed symmetry. Similarly, the fact that some layers, most notably the outmost layer, are significantly shorter (∼7 nm) could reflect a symmetry artifact, heterogeneity in protein position, and/or the presence of CA, which is smaller but ∼20× less abundant than RuBisCO according to our mass spectrometry data (see Table S1).

**Figure 6:**
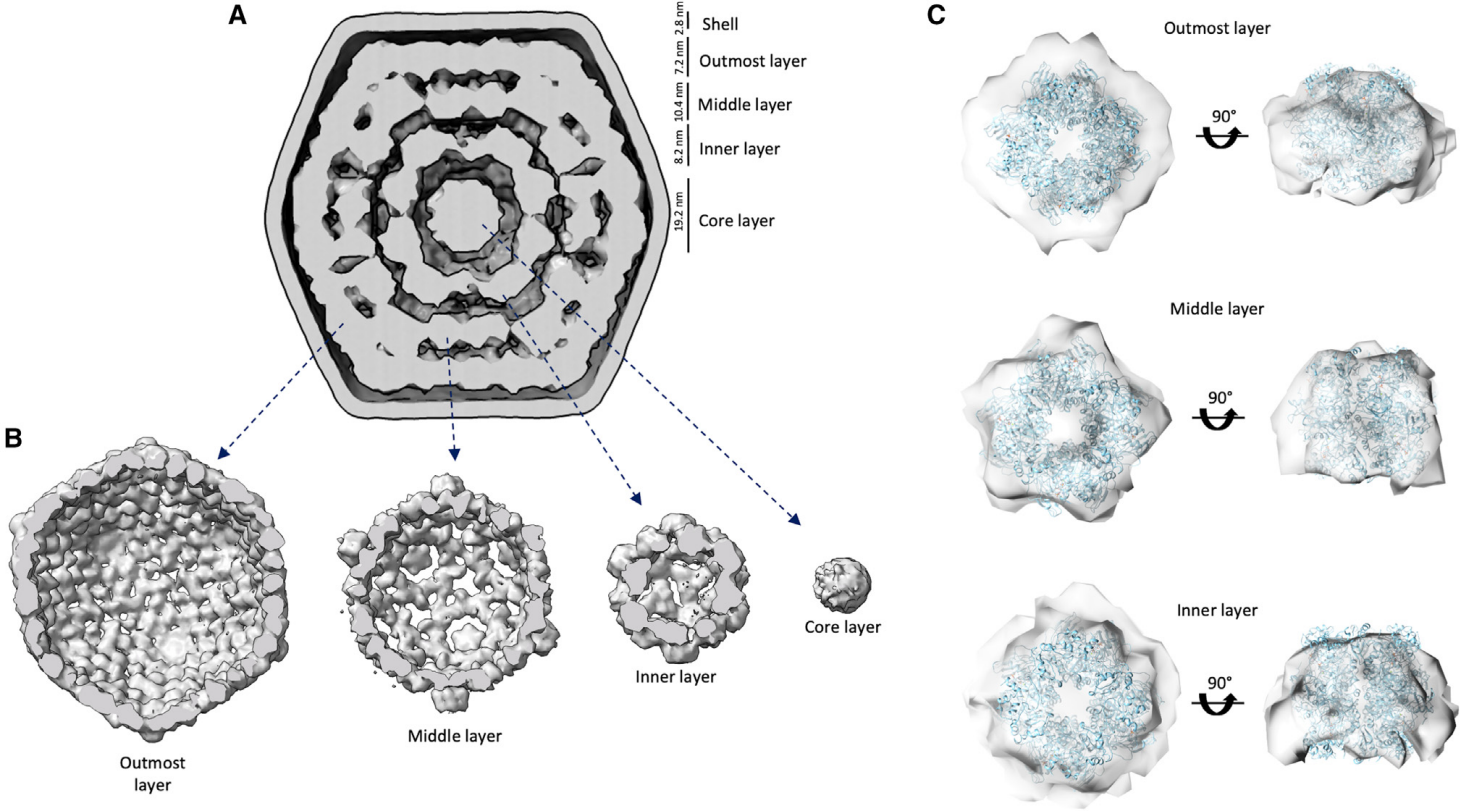
Internal density of the α-carboxysome structure (A) Slab through the overall density, revealing the different internal layers. The height of each layer is indicated. (B) Individual maps for each layer, obtained from selective masked refinement of the particles used in (A). (C) RuBisCO structure fitted into the cryo-EM maps generated for the internal density of the carboxysome. The quality of the fit for the outmost layer (top), middle layer (middle), and inner layer (bottom) are shown from the top and side. See also Figures S5 and S7.

**Figure 7:**
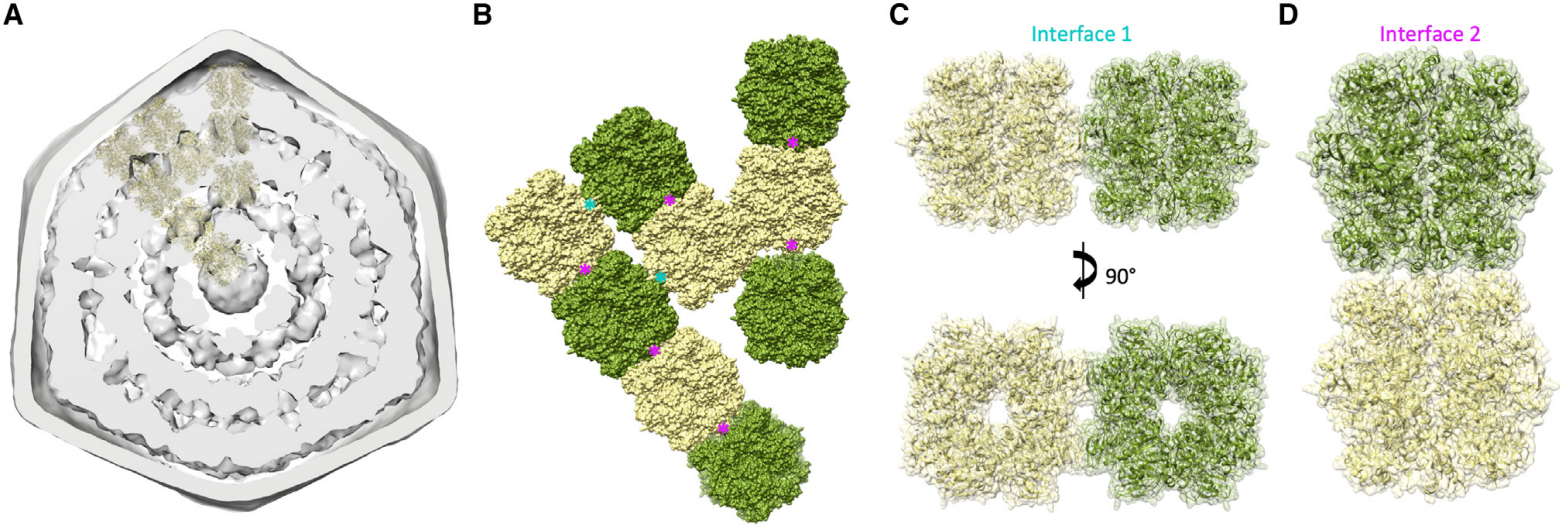
Internal arrangement of proteins within the α-carboxysome (A) Slab section of the α-carboxysome electron potential map, with nine RuBisCO complexes fitted in the internal density, in cartoon representation. The height of the complex fits the width and features of the internal density. (B) Surface representation of nine RuBisCO complexes from the internal organization model, in surface representation, and colored alternatively in yellow and green. Two distinct inter-RuBisCO interfaces are present, indicated with a cyan and magenta star, respectively. (C and D) Cartoon representation of two adjacent RuBisCO molecules forming the lateral (C) and longitudinal (D) interfaces, shown in cartoon through the transparent surface. The lateral contacts occur through loops in the CbbL subunit, while the longitudinal contacts are mediated by two helices in CbbL and CbbS. See also Figure S7, and Video S3.

The thickness of each layer, and the presence of features that are compatible with RuBisCO, allowed us to manually place individual complexes in the corresponding density (Figure 6C), leading to an atomic model of its internal organization within the carboxysome (Figure 7A; Video S3). In this model, RuBisCO forms concentric layers, and we were able to fit ∼300 RuBisCO within the internal density (4 in the core layer, 32 in the inner layer, 72 in the middle layer, and 192 in the outmost layer), roughly comparable with previous estimates.^43^ Particularly in the middle and outermost layers, gaps with thinner densities are present between RuBisCO molecules, which were not accounted for in our model. It is likely that these gaps accommodate CsoS2 and/or CA proteins; however, the intrinsically disordered structure of CsoS2 and the much smaller size of CA (compared with RuBisCO) did not permit us to model them within the densities.

Video S3. Structural model of the internal carboxysome organization, related to Figure 7

Our model of the α-carboxysome internal organization shows two RuBisCO interfaces (Figure 7B). The first interface corresponds to the contacts between RuBisCO proteins within the same layer and involves interactions on the lateral side of RuBisCO (Figure 7C). This interaction is mainly mediated via contacts in the variable loop region of the large subunit CbbL (Figure S8), where the CsoS2 N terminus has been proposed to bind,^40^ which awaits further validation. A second interface is formed by the interaction between RuBisCO proteins across the concentric layers in a top-to-bottom configuration (Figure 7D). In this case, the contacts appear to be largely mediated by two helices in the small subunit CbbS (Figure S8), although again, the limited resolution does not allow us to unambiguously resolve this. We note that both interfaces correspond to highly conserved regions of the RuBisCO proteins (Figure S8). This is most evident for the small subunit, where several residues in the top-to-bottom interface are conserved across organisms, and their conservation could indicate a common role in higher-order oligomerization.

## Discussion

In this study, we present a single-particle cryo-EM analysis of an intact α-carboxysome, purified from endogenous sources. Notably, we report the structure of its RuBisCO to 2.9 Å and observe the presence of unattributed densities on one side, suggesting that another protein is bound to some of the complexes. Using multistep classification, we obtained low-resolution maps of the icosahedral shell and of the internal cargo organization, which allows us to propose an atomic model for their respective architecture, through integrative modeling. Collectively, this work provides insights into the architecture of BMCs and their internal organization.

We chose the *Cyanobium* α-carboxysome as a model system in this study because its structure appears more homogeneous compared with other BMCs studied,^36^^,^^41^^,^^42^^,^^43^^,^^44^^,^^45^^,^^46^^,^^56^ as demonstrated in this (Figure S1) and previous studies.^29^^,^^48^ Our results demonstrate that its shell exhibits an icosahedral symmetry, albeit variable in shape and size, ranging from 119 to 123 nm in diameter (Figures 2C and S6). It confirms the common icosahedral architecture of carboxysomes in different species, as observed previously.^42^^,^^43^^,^^44^

The model of the internal RuBisCO organization within the α-carboxysome highlights four concentric layers of cargo enzymes and two main forms (side by side and top to bottom) of RuBisCO-RuBisCO interfaces (Figures 6 and 7). In contrast, recent work using cryoelectron tomography showed that in a distinct α-carboxysome from *Halothiobacillus neapolitanus*, RuBisCO forms filaments instead of concentric layers.^51^ Similarly, a recent cryoelectron tomography analysis of the same *Cyanobium* carboxysome samples provided similar insights, and largely achieved similar conclusions (most notably on the RuBisCO organization within carboxysomes).^57^ In those filaments, the interface is highly similar to one of the interfaces identified in our model. This strongly suggests that despite the diversity of α-carboxysome species, this top-to-bottom interaction is likely a conserved feature of RuBisCO-RuBisCO association. This conserved interaction is reminiscent of the recent discovery that many metabolic enzymes, such as CTP (cytidine triphosphate) synthase and IMPDH (inosine-5′-monophosphate dehydrogenase), are able to form higher-order assemblies to regulate their activities.^58^ Whether the RuBisCO assembly patterns inside the carboxysome could modulate RuBisCO activity merits further investigation. Moreover, it is likely that these filaments aid in the assembly and encapsulation of the shell in collusion with CsoS2. In comparison, RuBisCO enzymes in β-carboxysomes form paracrystalline arrays and exhibit relatively denser packing.^36^^,^^46^ The discrepancy in the internal organization and copy numbers of RuBisCO within α- and β-carboxysomes sheds light on their different assembly pathways and encapsulation mechanisms.

The low resolution of the α-carboxysome map reported here is partly due to the intrinsic heterogeneity and structural plasticity of natural carboxysome structures and internal RuBisCO packing. Given their dynamic and fast assembly, BMC structures are morphologically heterogeneous and vary in size and shape in their native host cells.^2^ It has also been shown that the abundance of individual proteins in the β-carboxysome and the size of β-carboxysomes in cyanobacteria is dynamically regulated in response to changing growth conditions.^59^ Moreover, the β-carboxysome shell appeared to be mechanically softer than virus capsids, highlighting the flexible nature of the shell architecture.^36^ The structural plasticity of BMCs also occurred in protein-protein interactions, such as dynamic self-assembly and correlation between shell protein paralogs to form specific protein assemblies and hetero-oligomers in BMCs.^15^^,^^53^^,^^54^^,^^60^ Consistently, our co-evolution analysis suggests that CsoS1A, CsoS1E, CsoS4A, and CsoS4B may form specific assemblies, in which CsoS4A and CsoS4B pentamers sit at the shell vertices, surrounded by CsoS1E proteins that then interact with CsoS1A hexamers (Figure 5B). It also suggests that the α-carboxysome shell paralogs CsoS1A and CsoS1E, as well as CsoS4A and CsoS4B, are prone to form hetero-oligomers (Table S2; Figure 4), as characterized in β-carboxysomes, which could function as a general mechanism for governing the passage of metabolites across the carboxysome shell. These flexible interactions may play vital roles in BMC shell assembly and permeability.

The power of single-particle cryo-EM should make it possible to determine the structure of an intact carboxysome at near-atomic resolution. However, there remains multiple practical challenges for this.^61^ Because of the structural heterogeneity mentioned above, a very large number of particles will be required; due to the distinct symmetry between the shell and internal layers, ideally no symmetry would be applied, again increasing the number of particles required for structure determination to high resolution. Additionally, the size of the complex necessitates collecting data with a large field of view, both limiting the attainable resolution and the throughput of data collection. Nonetheless, with the most recent wide-field direct-electron cameras,^62^ and with improved automation in data acquisition, this will likely be obtainable in the near future. We also emphasize that tomography approaches will also provide important new insights on the structural diversity of these complexes. While likely not reaching high-resolution structural information, tomography will notably be key to identify and characterize assembly intermediates and will provide a very important tool to understand the heterogeneous nature of carboxysomes.

Recently, extraordinary advances have been made in the acquisition of high-resolution characterization of synthetic BMC minishells.^18^^,^^19^^,^^20^^,^^52^^,^^63^ These synthetic shells, with minimal components, exhibit more homogeneous structures and lack any of the internal enzymes, thereby facilitating the alignment of the particles. In contrast, our study on the intact α-carboxysome structure provides insights into the carboxysome assembly as well as the diversity of BMC architectures and protein compositions. Further characterizations are expected to address how CsoS2 assists with the association of the outer layer of RuBiCO and shell proteins, how CsoS1D and CA are organized within the native α-carboxysome, and how the internal packing of RuBisCO enzymes is physiologically regulated.

## STAR★Methods

### Key resources table

**Table:** 

REAGENT or RESOURCE	SOURCE	IDENTIFIER
**Bacterial and virus strains**		

*Cyanobium* sp. PCC 7001	This study	https://img.jgi.doe.gov/cgi-bin/m/main.cgi?section=TaxonDetail&page=taxonDetail&taxon_oid=647533126

**Deposited data**		

Map of the carboxysome shell	This study	14379
Map of the carboxysome outermost layer	This study	14382
Map of the carboxysome middle layer	This study	14381
Map of the carboxysome innermost layer	This study	14380
Map of the carboxysome core layer	This study	14377
Map of RuBisCO with D4 symmetry	This study	14385
Coordinates of RuBisCO with D4 symmetry	This study	7YYO
Map of RuBisCO with C1 symmetry	This study	15409
Coordinates of RuBisCO with C1 symmetry	This study	8CMY

**Software and algorithms**		

CryoSPARC	Punjani et al., Nature Methods, 2017^64^	https://cryosparc.com/
Phenix	Afonine et al., ACTA D, 2018^65^	https://phenix-online.org/
ChimeraX	Pettersen et al., Protein Sci, 2021^66^	https://www.cgl.ucsf.edu/chimerax/
PyMol	The PyMOL Molecular Graphics System, Version 2.0 Schrödinger, LLC.	https://pymol.org/2/
AlphaFold	Jumper et al., Nature, 2021^67^	https://colab.research.google.com/github/sokrypton/ColabFold/blob/main/beta/AlphaFold2_advanced.ipynb
RaptorX	Jing et al., Methods Mol Biol, 2020^68^	http://raptorx.uchicago.edu/

### Resource availability

#### Lead contact

Further information and requests for resources and reagents should be directed to and will be fulfilled by the lead contact, Julien Bergeron (julien.bergeron@kcl.ac.uk).

#### Materials availability

This study did not generate new unique reagents.

### Experimental and model subject details

*Cyanobium* sp. PCC 7001 strains were cultured in BG-11 medium as described in the corresponding STAR Methods.

### Method details

#### Cyanobacterial strain growth and carboxysome purification

*Cyanobium* sp. PCC 7001 (Pasteur Culture Collection of Cyanobacteria, PCC) cells were grown in 4 L of BG-11 medium under constant illumination at 30°C with constant stirring and bubbling with air. Carboxysomes were purified as described previously with modifications. Cells were collected by centrifugation (6000 g, 10 min) and resuspended in TEB buffer (5 mM Tris-HCL, pH 8.0, 1 mM EDTA, 20 mM NaHCO_3_) with additional 0.55 M mannitol and 60 kU rLysozyme (Sigma-Aldrich, United States). Cells were then incubated overnight (20 h) with gentle shaking at 30°C in the dark, and were collected via centrifugation (6000 g, 10 min). Cells were placed on ice and resuspended in 20 mL ice-cold TEB containing an additional 5 mL 1 μm Silicone disruption beads. Cells were broken via bead beating for 8 mins in one-minute intervals of vortex, and 1 min on ice. Broken cells were separated from the beads, and the total resuspension volume was increased to 40 mL with TEB buffer containing an additional 4% IGEPAL CA-630 (Sigma-Aldrich, United States) were mixed on a rotating shaker overnight at 4°C. Unbroken cells were pelleted via centrifugation at 3,000 g for 5 mins, and the supernatant was centrifuged at 40,000 g for 20 mins. The pellet was then resuspended in 40 mL TEMB containing 4% IGEPAL CA-630 and centrifuged again at 40,000 x g for 20 mins. The resulting pellet was then resuspended in 2 mL TEB + 10mM MgCl_2_ (TEMB) (5 mM Tris-HCL, pH 8.0, 1 mM EDTA, 10 mM MgCl_2_, 20 mM NaHCO_3_) and centrifuged at 5000 x g for 5 mins before loading onto a 20-60% (v/v) sucrose gradient in TEMB buffer. Gradients were then centrifuged at 105,000 g for 60 mins at 4°C; the milky band at the 40%-50% interface was collected, diluted in 10 mL TEMB buffer and centrifuged again at 105,000 g for 60 mins. The final carboxysome pellet was then resuspended in 150 μL TEMB for the following structural and biochemical analysis.

#### SDS-PAGE and immunoblot analysis

Isolated carboxysomes were diluted to 5 mg⋅mL^-1^ and denatured using 4X Bromophenol blue buffer (Fisher Scientific, United States). The samples were heated at 95°C for 10 mins, and insoluble debris was pelleted via short spin. Approximately 50 μg proteins were loaded onto 15% (v/v) denaturing SDS-PAGE gels and stained using Coomassie Brilliant Blue G-250 (ThermoFisher Scientific, UK). Immunoblot analyses were performed using anti-CbbL (1:10,000 diution, Agrisera, AS03 037, Sweden), anti-CsoS1 from *H. neapolitanus* (1:5000 dilution, Agrisera, AS14 2760, Sweden), and horseradish peroxidase-conjugated goat antirabbit immunoglobulin G secondary antibody (1:10,000 dilution, Agrisera AS101461, Sweden). Images were taken using a Quant LAS 4000 platform (GE Healthcare Life Sciences, USA).

#### RuBisCO assay

RuBisCO activities of isolated carboxysomes were determined as described previously with minor modifications.^25^^,^^31^^,^^36^ Isolated α-carboxysomes were diluted to 0.5 mg mL^-1^ in (100 mM EPPS, pH 8.0; 20 mM MgCl_2_) and 5 μL was added to scintillation vials containing NaH^14^CO_3_ with a range of concentrations (1.5-48 mM). and incubated at 37 °C for 2 mins before the addition of D-ribulose 1,5-bisphosphate sodium salt hydrate (RuBP, Sigma Aldrich, US) final concentration 0.04 mM. The reaction was carried out for 5 mins before being terminated by adding 2:1 by volume 10% formic acid. Samples were dried for at least 30 mins at 95 °C to remove unfixed ^14^C before re-suspending the fixed ^14^C pellets with ultra-pure water and adding 2 mL of scintillation cocktail (Ultima Gold XR, PerkinElmer, US). Radioactivity measurements were then performed using a scintillation counter (Tri-Carb, PerkinElmer, US). Raw readings were used to calculate the amount of fixed ^14^C, and then converted to the total carbon fixation rates. RuBisCO activity. Data are presented as mean ± standard deviation (SD) based on three biological replicates isolated from independent culture batches, and were analyzed using OriginPro 2020b (OriginLab, Massachusetts, USA).

#### Mass spectrometry analysis

The isolated α-carboxysome samples were washed with PBS buffer. Rapigest was added to a final concentration of 0.05% (w/v) into the sample for 10-min incubation at 80°C. The sample was then reduced with dithiothreitol (3 mM, final concentration) for 10 mins at 60°C, alkylated with iodoacetamide (9 mM, final concentration) for 30 min at room temperature in the dark, followed by digestion with trypsin at 37°C overnight. Digestion was terminated with 1 μL of trifluoroacetic acid (TFA). Data-dependent LC-MS/MS analysis was conducted on a QExactive quadrupole-Orbitrap mass spectrometer coupled to a Dionex Ultimate 3000 RSLC nano-liquid chromatograph (Hemel Hempstead, UK). A 2 μL sample digest was loaded onto a trapping column (Acclaim PepMap 100 C18, 75 μm × 2 cm, 3 μm packing material, 100 Å) in 0.1% TFA, 2% acetonitrile H_2_O, and set in line with the analytical column (EASY-Spray PepMap RSLC C18, 75 μm × 50 cm, 2 μm packing material, 100 Å). Peptides were eluted using a linear gradient of 96.2% buffer A (0.1% formic acid):3.8% buffer B (0.1% formic acid in water:acetonitrile 80:20, v/v) to 50% buffer A:50% buffer B over 30 mins at 300 nL min^-1^. The mass spectrometry analysis was operated in DDA mode with survey scans between *m*/*z* 300-2000 acquired at a mass resolution of 70,000 (FWHM) at *m*/*z* 200. The maximum injection time was 250 ms, and the automatic gain control was set to 1e^6^. Fragmentation of the peptides was performed by higher-energy collisional dissociation using a normalized collision energy of 30%. Dynamic exclusion of *m*/*z* values to prevent repeated fragmentation of the same peptide was used with an exclusion time of 20 seconds.

#### Thin-section electron microscopy

Cyanobacterial cell cultures were pelleted by centrifugation (6,000 g, 10 min) and processed for thin section using a Pelco BioWave Pro laboratory microwave system. The cells are first fixed with 2.5% glutaraldehyde in 0.1 M sodium cacodylate buffer at pH 7.2 using two steps of 100W. After agarose embedding, samples were then stained with 2% osmium tetroxide and 3% Potassium Ferrocyanide using three steps of 100W. The osmium stain was set using 1% thiocarbohydrazide and 2% osmium tetroxide. The samples were stained with 2% uranyl acetate, prior to dehydration by increasing alcohol concentrations (from 30 to 100%) and resin embedding. Thin sections of 70 nm were cut with a diamond knife and poststained with 3% lead citrate.

#### Negative-stain TEM grid preparation and screening

Isolated α-carboxysome samples were immobilized onto the glow-discharged grids and then were stained with 2% uranyl acetate. EM imaging was conducted using an FEI Tecnai G2 Spirit BioTWIN transmission electron microscope equipped with a Gatan Rio 16 camera.

#### Cryo-EM grid preparation and data collection

For the structural characterisation of RuBisCO, 3 μL aliquots of purified α-carboxysomes at a concentration of ∼1 mg⋅mL^-1^ were applied to Graphene Oxide coated, 300 mesh, 2/2 μm hole/spacing, holey carbon grids (EMR). A Leica EM GP Automatic Plunge Freezer (Leica) was used to plunge freeze the sample, blotting for 3-6 s. Cryo-EM data was collected with a 300 kV Titan Krios TEM, equipped with a Falcon 3 direct electron detector (Thermo Fisher) operated in linear mode. 4593 micrographs were collected using the EPU software (Thermo Fisher) with a pixel size of 1.11 Å pix^-1^, a total dose rate of 30 e^-^ Å^-2^, and 44 fractions per micrograph. The defocus range was -0.5 to -1.5 μm.

For structural characterisation of the intact α-carboxysome complex, 3 μL aliquots of purified sample at a concentration of 3 mg⋅mL^-1^ were applied to Graphene Oxide coated grids, 300 mesh, 2/2 μm hole/spacing, holey carbon grids (EMR). A Leica EM GP Automatic Plunge Freezer (Leica) was used to plunge freeze, blotting for 6 s. Cryo-EM data were collected with a 300 kV Titan Krios TEM with a Falcon 3 direct electron detector (FEI) operated in counting mode. 5429 micrographs were collected using EPU software (Thermo Fisher) with a pixel size of 2.23 Å pix^-1^ with a total dose rate of 29.7 e^-^ Å^-2^ with 33 frames per micrograph. The defocus range was -1.0 to -2.2 μm.

### Quantification and statistical analysis

#### Mass spectrometry data analysis

The raw data files were imported into Progenesis QI for Proteomics v4 (Nonlinear Dynamics, Newcastle upon Tyne UK, a Waters Company). The chromatograms are aligned and normalised prior to label-free quantification. Peptide identification was performed by Mascot (v2.8, Matrix Science, UK) against the Uniprot reference *Cyanobium* sp. PCC 7001 database (UP000003950, 2771 proteins). A precursor mass tolerance of 10 ppm and a fragment ion mass tolerance of 0.01 Da were applied with the dynamic modification of Oxidation (M) and with the static modification of carbamidomethylation (C).

#### Cryo-EM data processing

All the cryo-EM data processing steps were carried out in CryoSPARC^64^ v.3.1.0.

For the RuBisCO structure, automated particle picking was initially used, leading to a dataset of ∼ 2,800,000 particles. 2D classification was employed to select particles that clearly correspond to RuBisCo, leading to a final set of 131,356 particles. 3D refinement was performed with these, with D4 symmetry, converging to a map at 2.87 Å resolution. The same set of particles was also refined without symmetry imposed, leading to a second map at 3.79 Å resolution.

For intact carboxysomes, 131 particles were manually picked from selected micrographs to generate 2D classes subsequently used for template picking for the entire dataset. A total of 15,545 particles were picked and extracted using a 700x700 pixels box. After multiple rounds of 2D classification 8,701 particles from the best 2D classes were selected and used to generate an initial model. Particles were downsampled to a box size of 168x168 pixels for 3D classifications and reconstructions. A reconstruction of the entire carboxysome was generated in I symmetry. Masked classifications of the shell were carried out with C1 symmetry to give a reconstruction at 19 Å resolution. Heterogeneous refinements of the carboxysome shell used for model building were carried out with I symmetry to give reconstructions of ∼18 Å.

#### Modelling and co-evolution analysis

Atomic models of the CsoS1A and CsoS1E hexamers, the CsoS4A and CsoS4B pentamers, and the CsoS1D trimer were generated with AlphaFold2 (ColabFold).^64^^,^^69^ The co-evolution analyses were performed using the RaptorX server,^69^ with contact probabilities > 0.5 considered to be significant.

To build the *Cyanobium* sp. PCC 7001 RuBisCO structure, an initial atomic model was built for both CbbL and CbbS with AlphaFold2 (ColabFold),^67^^,^^70^ and 8 copies of each were placed at their respective location on the EM map. The coordinates for the substrate and Mg ion were added manually, and the termini without visible density were deleted. The model was then subjected to real-space refinement in Phenix.^65^

The difference map was calculated by first generating a volume of the RuBisCO structure, and then subtracting this volume from the C1 reconstruction, in ChimeraX.^66^

To generate the atomic model of the shell, a CsoS4a pentamer was placed in one corner of the map icosahedron, using the orientation reported previously in the structure of the β-carboxysome synthetic shell^20^ to determine the outward face. Five copies of the CsoS1E hexamer were placed around it, again using the β-carboxysome structure to determine the outward face. Additional copies of the CsoS1A hexamers were next placed manually, forming additional continuous layers around until five faces of the carboxysome shell were complete. The model was manually curated in Chimera^71^ and used for global energy minimisation refinement in Phenix.

For the internal density, copies of the *Cyanobium* sp. PCC 7001 RuBisCO structure were placed in regions of the map of the different shells, and fitted manually in Chimera. If major clashes were observed between adjacent molecules, that with the less optimal fit to the density was removed.

All structural figures were generated in either PyMol,^72^ Chimera, or ChimeraX.

## Data Availability

The structure of the *Cyanobium* sp. PCC 7001 RuBisCO enzyme with D4 symmetry has been deposited to the PDB (PDB: 7YYO), and the corresponding 2.9 Å cryo-EM map was deposited to the EMDB (EMDB: 14385). The structure obtained without imposing any symmetry has been deposited to the PDB (PDB: 8CMY), and the corresponding 3.8 Å map was also deposited to the EMDB (EMDB: 15409). The maps of the carboxysome shell, and of each individual internal layer, have been deposited to the EMDB (EMDB: 14379, 14382, 14381, 14380, 14377). This paper does not report original code. Any additional information required to reanalyse the data reported in this paper is available from the lead contact upon request.

## Supplemental information

Document S1. Figures S1–S8 and Tables S1 and S2

Document S2. Article plus supplemental information
