# Supplementary material for: Single-particle cryo-EM analysis of the shell architecture and internal organization of an intact α-carboxysome
Source: Structure. 2023 Jun 1;31(6):677–688.e4. doi: 10.1016/j.str.2023.03.008 (PMC10689251; doi:10.1016/j.str.2023.03.008)
Supplement: Document S1. Figures S1–S8 and Tables S1 and S2 [file mmc1.pdf]

Structure, Volume *31*

Supplemental Information

Single-particle cryo-EM analysis of the shell architecture and internal organization of an intact α-carboxysome

Sasha L. Evans, Monsour M.J. Al-Hazeem, Daniel Mann, Nicolas Smetacek, Andrew J. Beavil, Yaqi Sun, Taiyu Chen, Gregory F. Dykes, Lu-Ning Liu, and Julien R.C. Bergeron

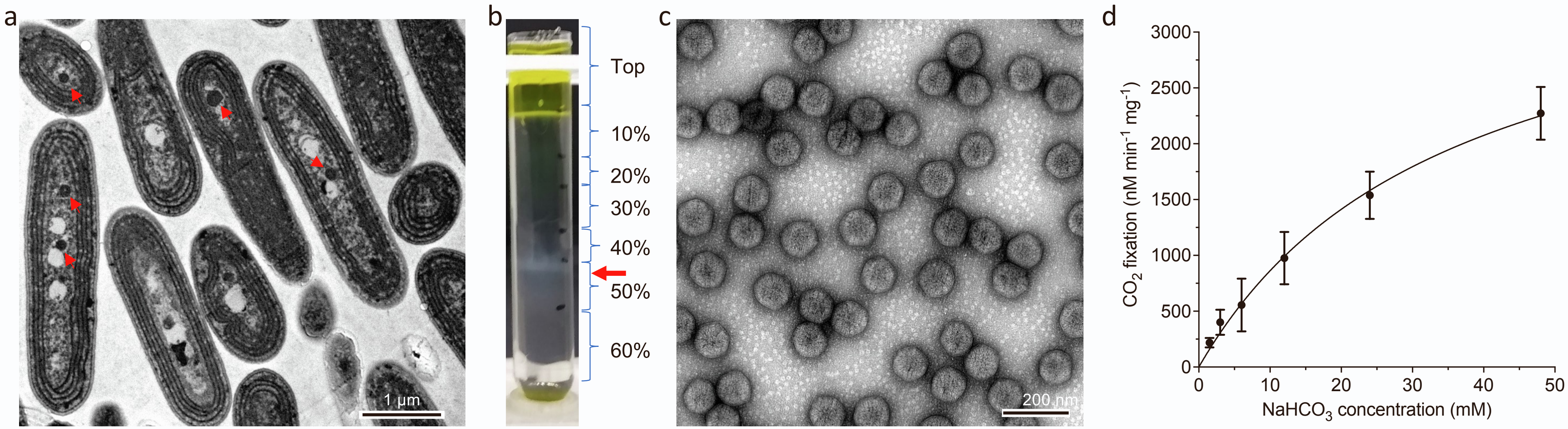

Figure S1: Isolation and purification of the *Cyanobium* α-carboxysome. Related to Figure 2. (a) Thin section of cultured cells, with carboxysomes indicated with red arrows. **(b)** Sucrose gradient from the purification process. Carboxysome-enriched samples are indicated in red arrow. **(c)** Negative-stain micrograph of purified carboxysome complexes. RuBisCO molecules, presumably from broken carboxysome complexes, can be seen in the background. **(d)** RuBisCO activity assay from the purified α-carboxysomes, demonstrating that they are functional for carbon fixation. Data are presented as mean ± standard deviation (SD) based on three biological replicates isolated from independent culture batches.

Protein	Score	Mass (Da)	Normalized amount (fmol)
**CbbL**	3376.57	53050	8682.5 ± 2435.3
**CsoS2**	5646.53	86750	1288.5 ± 194.5
**CSoS1A**	1661.34	10628	9654.3 ± 192.0
**CbbS**	505.73	13061	4659.1 ± 3110.8
**CsoSCA**	1771.19	61331	118.0 ± 18.0
**CsoS1E**	831.92	18563	181.1 ± 55.6
**CsoS4A**	104.78	10317	5.4 ± 8.8
**CsoS1D**	327.88	26073	1.6 ± 0.1
**CsoS4B**	145.98	8894	1.0 ± 1.7
**Bacterioferritin**	687.17	17782	88.7 ± 49.1
**HAM1**	451.01	20496	8.6 ± 5.2

Table S1: Proteomic results of isolated α-carboxysomes from *Cyanobium*. Related to Figure 2. The column of Normalized amount displays the amount of each of the carboxysomal proteins detected in isolated α-carboxysomes using mass spectrometry, normalized against the amount of the least abundant protein CsoS4B. Note: Mass spectrometry revealed the presence of bacterioferritins and purine NTP pyrophosphatases in the isolated α-carboxysome samples. Both genes encoding bacterioferritin (CPCC7001_1612) and purine NTP pyrophosphatase (CPCC7001_2175) are located within the α-carboxysome operon in the *Cyanobium* genome.

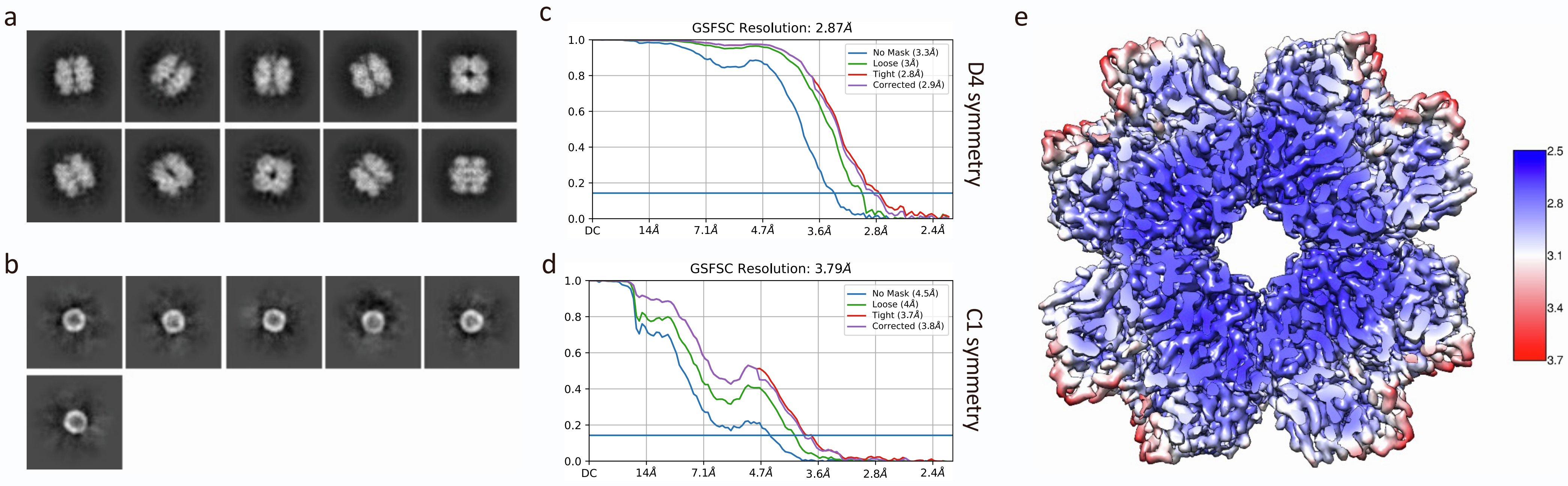

Figure S2: Cryo-EM reconstruction of the spilled RuBisCo. Related to Figure 3. (a), (b) 2D classification of the proteins spilled from broken α-carboxysomes. (a) A subset of particles formed highly-ordered 2D classes, whose size and overall shape matched that of RuBisCo. (b) A second sub-set of particles were smaller, and not well resolved. **(c), (d)** Half-map FSC curves for the RuBisCo structures, with D4 symmetry (c) and with C1 symmetry (d). **(e)** RuBisCo electron potential map colored by local resolution.

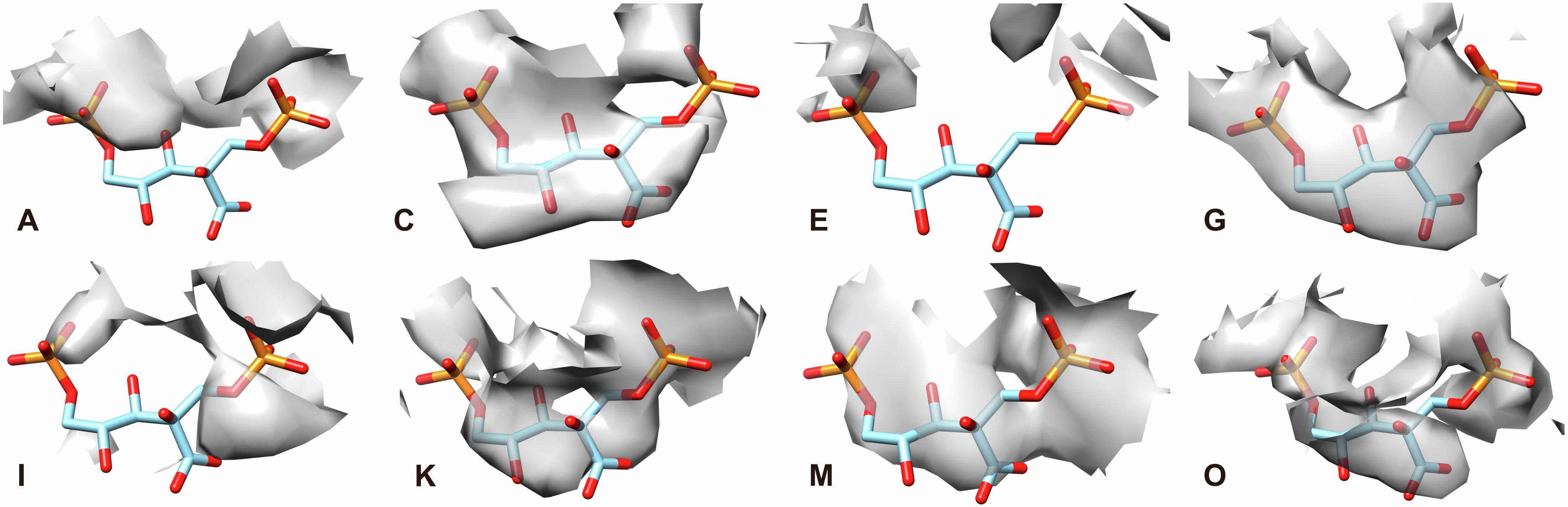

Figure S3: Substrate density in the C1 RuBisCO reconstruction. Related to Figure 3. The map density at the substrate position, as determined in the D4 map, is shown for all eight subunits. Density for the substrate (RuBP) can be seen for subunit G, partial density is observed for subunits C, I, K, M and O potentially indicative of the product (3PG). No density is present in subunits A and E. RuBP is modeled in all of the subunits for clarity.

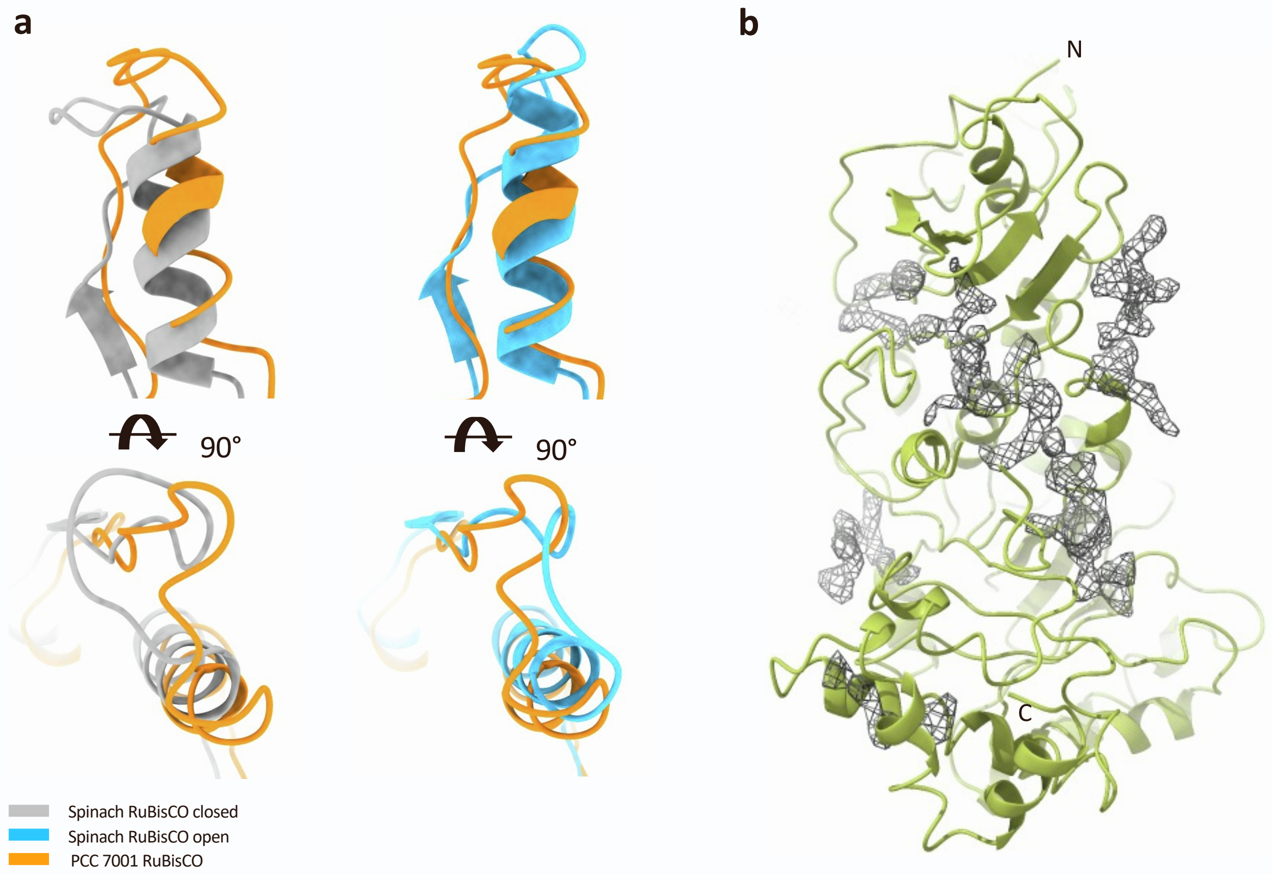

Figure S4: Analysis of the PCC7001 RuBisCO structure. Related to Figure 3. (a) Comparison of loop 6 position in *Cyanobium* sp. PCC7001 RuBisCO (orange) with closed (grey) and open (blue) spinach RuBisCO crystal structures, respectively. **(b)** Continuous density at the RuBisCO surface. The difference map between the D4 and C1 RuBisCO maps shown in mesh, with the corresponding atomic model. Density resembling an extended stretch of amino-acids is visible.

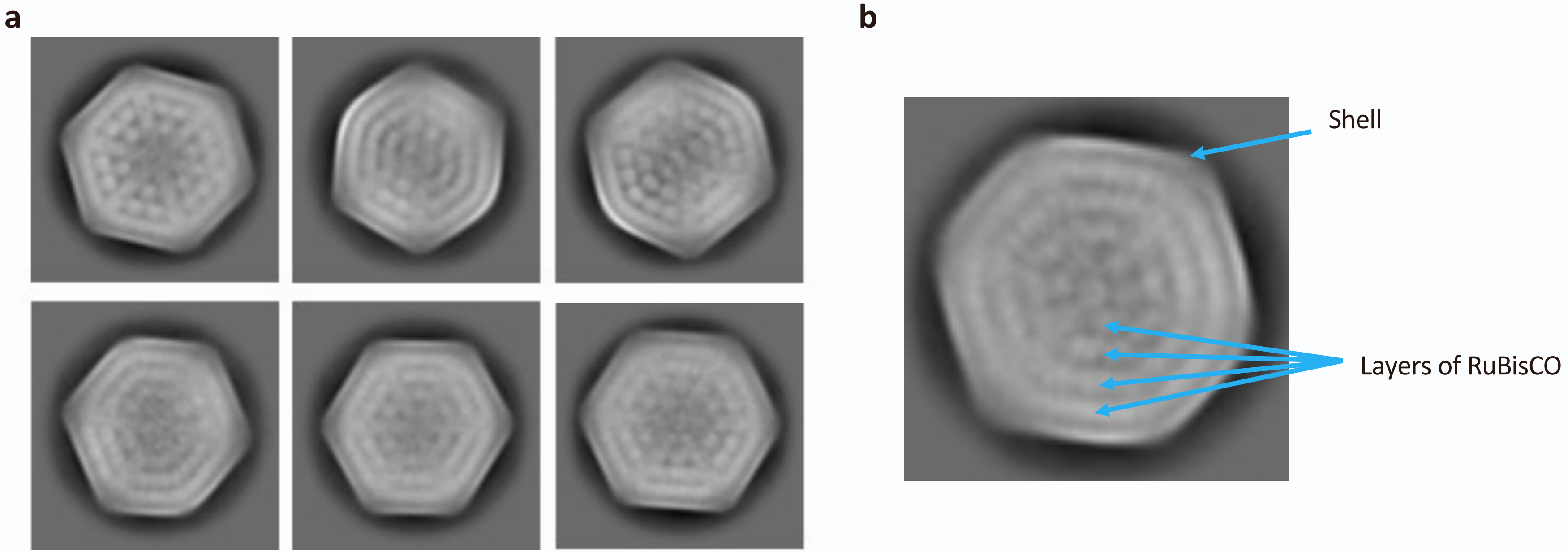

Figure S5: 2D classification of intact α-carboxysome particles. Related to Figures 5 and 6. (a) selected 2D classes of α-carboxysome complexes from the second dataset, demonstrating a rigid organization of the internal enzymes. **(b)** Close-up view of one such 2D class, with the localization of the shell, and internal RuBisCO layers.

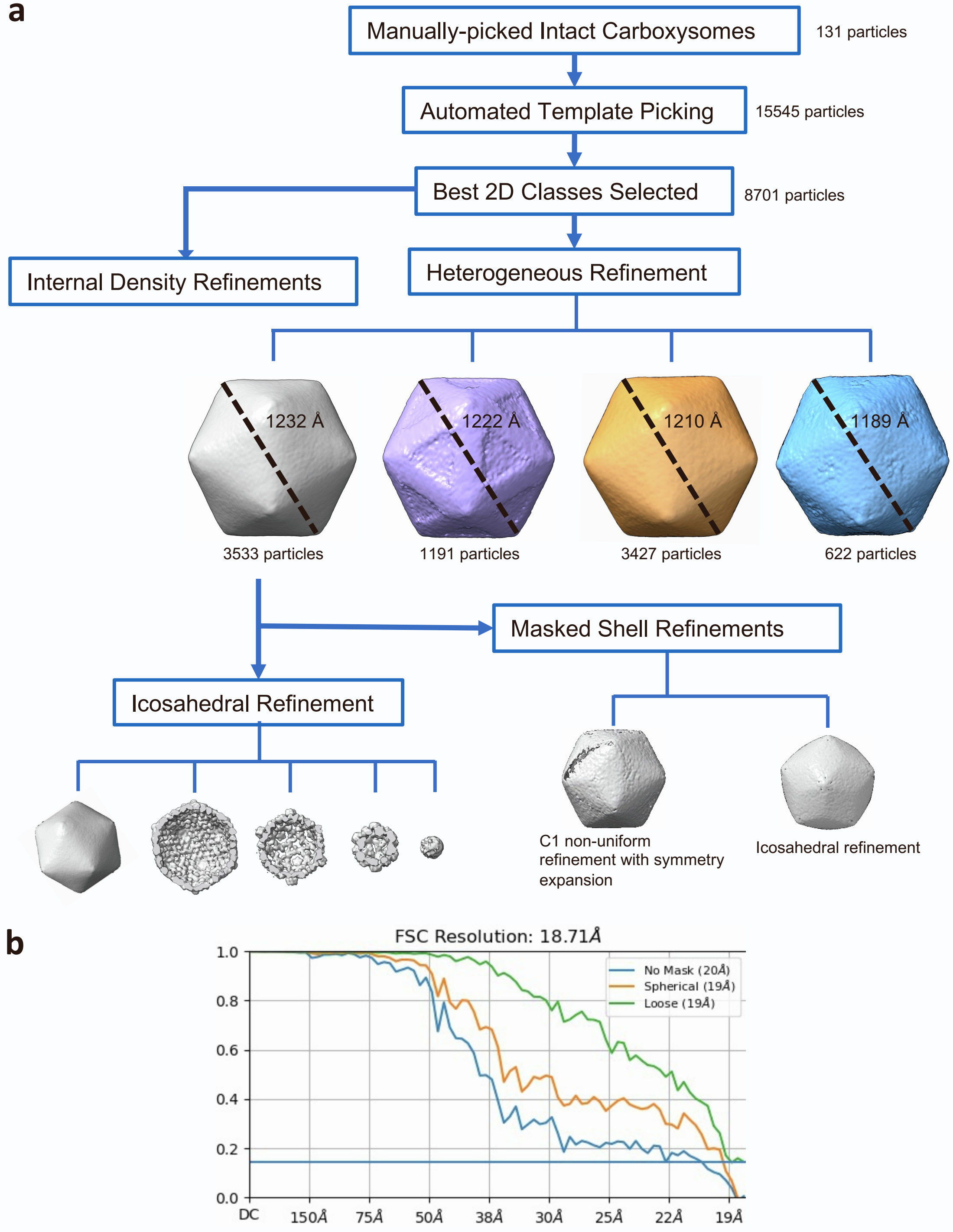

Figure S6: Cryo-EM processing pipeline including classification and refinement of the α-carboxysome shell. Related to STAR Methods. (a) The various steps used for the processing of the 2nd dataset are indicated. 3D classes of the shell particles. The for each class, the number of particles and the shell diameter are indicated. **(b)** FSC curve for the carboxysome shell map with icosahedral symmetry.

	CsoS1A	CsoS1D	CsoS1E	CsoS4A	CsoS4B	CsoS2
**CsoS1A**	/	0	8	1	3	14
**CsoS1D**	/	/	2	1	0	2
**CsoS1E**	/	/	/	4	6	12
**CsoS4A**	/	/	/	/	1	2
**CsoS4B**	/	/	/	/	/	1
**CsoS2**	/	/	/	/	/	/

Table S2: Co-evolution analysis of the shell proteins. Related to Figure 4. The number of co-evolving residues with a score > 0.5 for each protein pair is indicated.

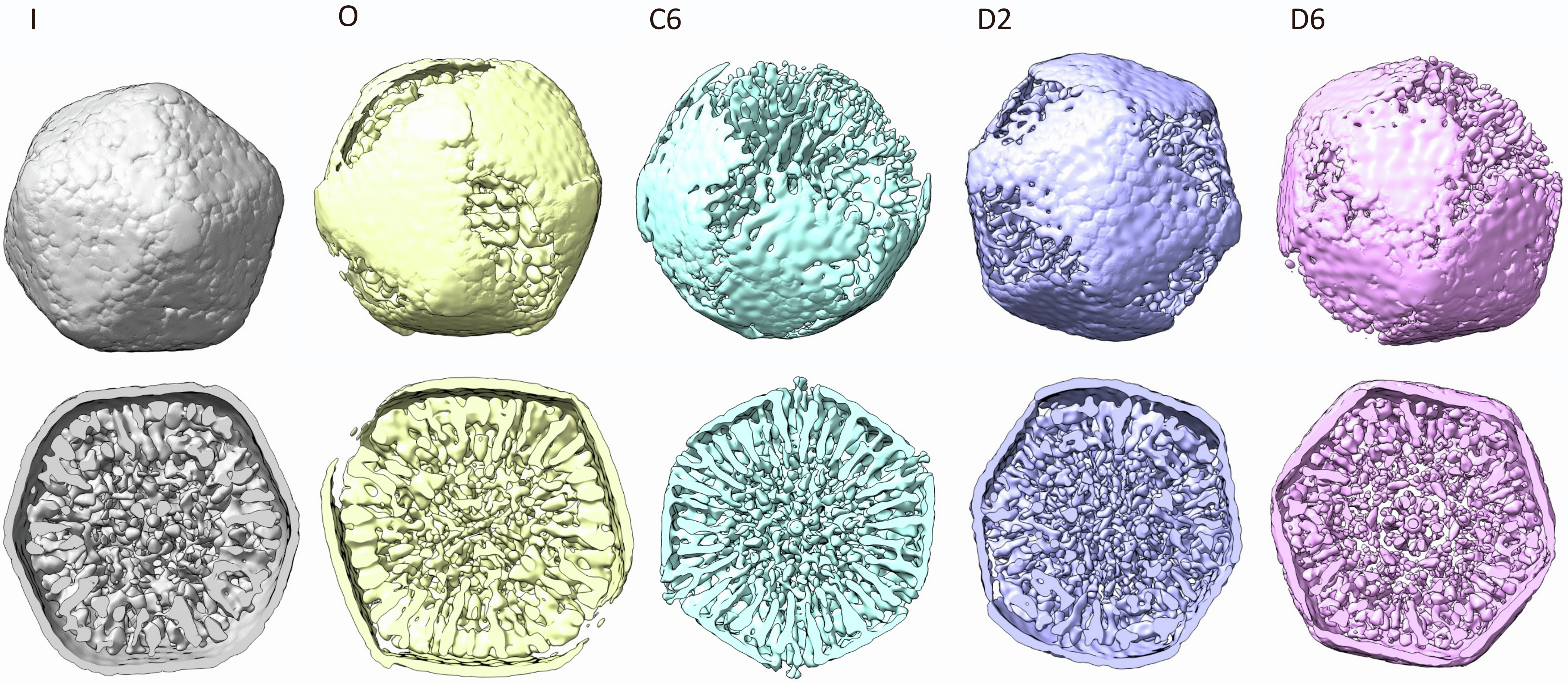

Figure S7: 3D refinement of intact α-carboxysome particles, with a range of symmetries. Related to Figure 6. For each symmetry used, the overall map, as well as a transversal section through the density to reveal the internal layers.

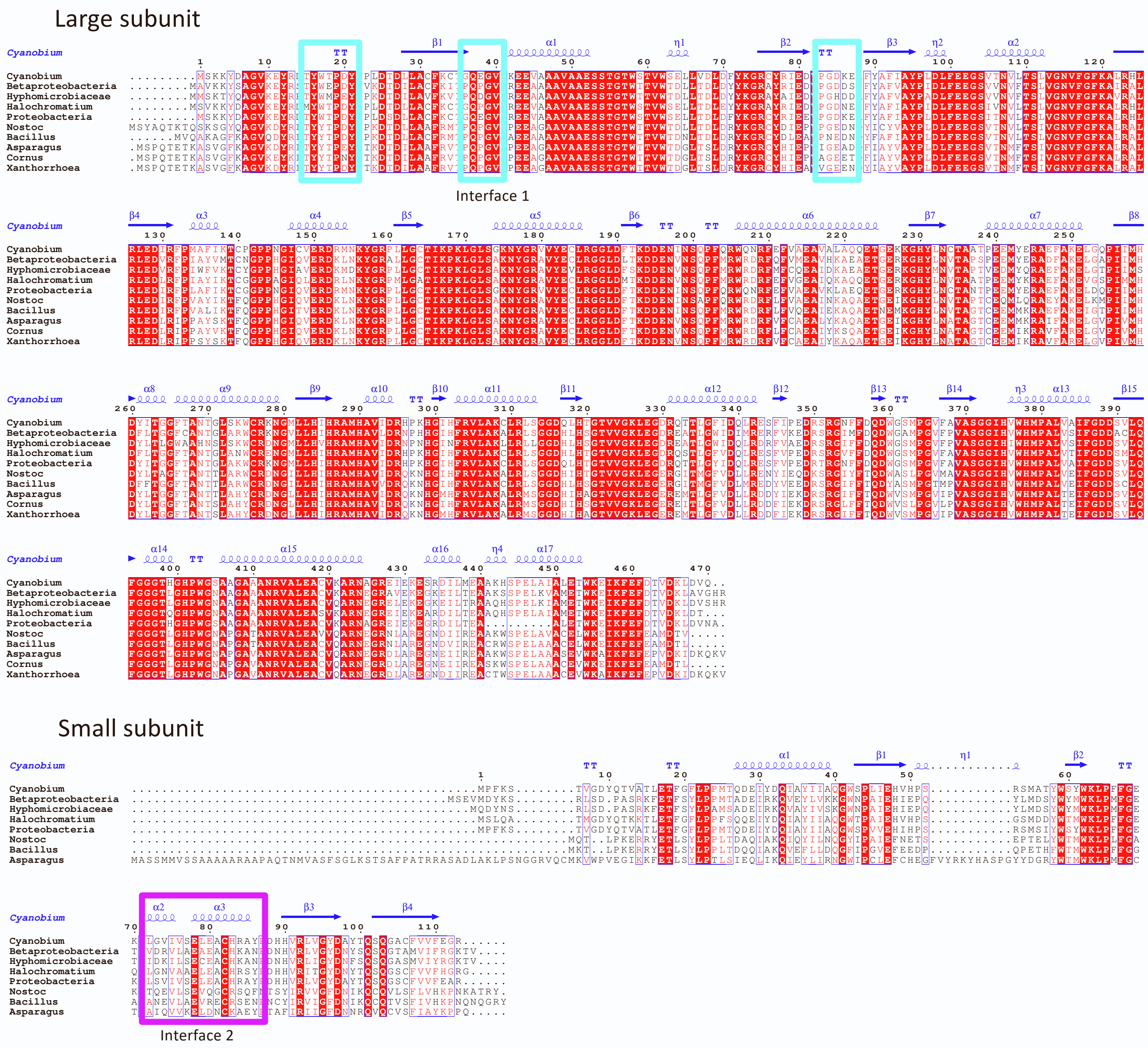

Figure S8: Multiple sequence alignments of the large and small RuBisCO subunits. Related to Figure 7. Conserved residues are boxed in red, similar residues are in red fonts. The secondary structure for the Cyanobium RuBisCO, reported here, is shown at the top. The lateral and longitudinal contacts observed between RuBisCO molecules within the intact carboxysome are indicated with cyan and magenta boxes, respectively.
